# *FANCM* mutation c.5791C>T is a risk factor for triple-negative breast cancer in the Finnish population

**DOI:** 10.1007/s10549-017-4388-0

**Published:** 2017-07-12

**Authors:** Johanna I. Kiiski, Anna Tervasmäki, Liisa M. Pelttari, Sofia Khan, Tuomo Mantere, Katri Pylkäs, Arto Mannermaa, Maria Tengström, Anders Kvist, Åke Borg, Veli-Matti Kosma, Anne Kallioniemi, Johanna Schleutker, Ralf Bützow, Carl Blomqvist, Kristiina Aittomäki, Robert Winqvist, Heli Nevanlinna

**Affiliations:** 1 Department of Obstetrics and Gynecology, University of Helsinki and Helsinki University Hospital, Helsinki, Finland; 2 Laboratory of Cancer Genetics and Tumor Biology, Cancer and Translational Medicine Research Unit, Biocenter Oulu, University of Oulu, Oulu, Finland; 3 Laboratory of Cancer Genetics and Tumor Biology, Northern Finland Laboratory Centre, NordLab, Oulu, Finland; 4 School of Medicine, Institute of Clinical Medicine, Pathology and Forensic Medicine, and Cancer Center of Eastern Finland, University of Eastern Finland, Kuopio, Finland; 5 Imaging Center, Clinical Pathology, Kuopio University Hospital, Kuopio, Finland; 6 School of Medicine, Institute of Clinical Medicine, Oncology, Kuopio, Finland; 7 Cancer Center, Kuopio University Hospital, Kuopio, Finland; 8 Division of Oncology and Pathology Department of Clinical Sciences Lund, Lund University, Medicon Village, Lund, Sweden; 9 BioMediTech Institute and Faculty of Medicine and Life Sciences, University of Tampere, and Fimlab Laboratories, Tampere, Finland; 10 Department of Medical Biochemistry and Genetics, Institute of Biomedicine, University of Turku, Turku, Finland; 11 Department of Medical Genetics, Tyks Microbiology and Genetics, Turku University Hospital, Turku, Finland; 12 Department of Pathology, University of Helsinki and Helsinki University Hospital, Helsinki, Finland; 13 Department of Oncology, University of Helsinki and Helsinki University Hospital, Helsinki, Finland; 14 Department of Clinical Genetics, University of Helsinki and Helsinki University Hospital, Helsinki, Finland; 15 Department of Obstetrics and Gynecology, Helsinki University Central Hospital (HUS), PO Box 700, 00029 Helsinki, Finland

**Keywords:** FANCM, Breast cancer, Triple-negative breast cancer, Familial breast cancer, DNA repair

## Abstract

**Purpose:**

The *FANCM* c.5101C>T nonsense mutation was previously found to associate with breast cancer in the Finnish population, especially among triple-negative cases. Here, we studied the prevalence of three other *FANCM* variants: c.5791C>T, which has been reported to predispose to familial breast cancer, and the c.4025_4026delCT and c.5293dupA variants recently identified in Finnish cancer patients.

**Methods:**

We genotyped the *FANCM* c.5791C>T mutation in 4806 invasive breast cancer patients, including *BRCA1/2* mutation negative familial cases and unselected cases, and in 2734 healthy population controls from four different geographical areas of Finland. The association of the mutation with breast cancer risk among patient subgroups was statistically evaluated. We further analyzed the combined risk associated with c.5101C>T and c.5791C>T mutations. We also genotyped 526 unselected ovarian cancer patients for the c.5791C>T mutation and 862 familial breast cancer patients for the c.4025_4026delCT and c.5293dupA variants.

**Results:**

The frequency of the *FANCM* c.5791C>T mutation was higher among breast cancer cases than in controls (OR 1.94, 95% CI 0.87–4.32, *P* = 0.11), with a statistically significant association with triple-negative breast cancer (OR 5.14, 95% CI 1.65–16.0, *P* = 0.005). The combined analysis for c.5101C>T and c.5791C>T carriers confirmed a strong association with breast cancer (OR 1.86, 95% CI 1.32–2.49, *P* = 0.0002), especially among the triple-negative patients (OR 3.08, 95% CI 1.77–5.35, *P* = 0.00007). For the other variants, only one additional c.4025_4026delCT carrier and no c.5293dupA carriers were observed.

**Conclusions:**

These results support the role of *FANCM* as a breast cancer susceptibility gene, particularly for triple-negative breast cancer.

## Introduction

Fanconi Anemia complementation group M (FANCM) is a multifunctional protein, interacting with several partners to activate the Fanconi anemia (FA) repair pathway. The pathway includes 19 associated proteins, which form multiprotein complexes to repair damaged DNA, especially stalled replication forks induced by interstrand cross-linking (ICL) agents [1, 2]. FA itself is a rare, recessively inherited genetic disorder causing congenital defects, hematologic abnormalities, and cancer predisposition [1].

Despite the nomenclature, FANCM does not appear to have a role in the FA disease, as the initial case, which led to the FANCM association with FA, also carried biallelic *FANCA* mutations. Furthermore, homozygous loss-of-function mutations in *FANCM* have been identified in individuals without any FA symptoms [3, 4]. However, FANCM has an undeniable role in the FA pathway, as it recruits other DNA damage response proteins to the sites of DNA lesions [4]. Several FA genes (*FANCD1/BRCA2*, *FANCN/PALB2*, *FANCJ/BRIP1*, *FANCO/RAD51C*, and *FANCS/BRCA1*) [5–11] are high- or moderate-risk breast or ovarian cancer susceptibility genes and previous studies have connected also heterozygous *FANCM* mutations with breast cancer predisposition [12–14].

We previously identified the *FANCM* c.5101C>T nonsense mutation (rs147021911, p.Gln1701*) in exon 20 by exome sequencing of germline DNA samples from 24 *BRCA1/2*-negative breast cancer patients and further genotyped it in a large series of Finnish breast cancer patients and healthy population controls [12]. The mutation was found to be associated with breast cancer with an odds ratio (OR) of 1.86 [95% confidence interval (CI) 1.26–2.75, *P* = 0.0018], especially among triple-negative breast cancer [TNBC; estrogen (ER) and progesterone (PR) receptor and HER2 negative] cases (OR 3.56, 95% CI 1.81–6.98, *P* = 0.0002). According to the ExAC [15] database this mutation is more common in Finland (carrier frequency ~1.8%) than in other European populations (carrier frequency ~0.3%).

Another, yet more rare *FANCM* mutation, c.5791C>T (rs144567652, p.Arg1931*) in exon 22, has been identified with exome sequencing of breast cancer patients [16]. Further analysis of familial breast cancer cases and healthy controls from several populations showed an association between the mutation and familial breast cancer (OR 3.93, 95% CI 1.28–12.11, *P* = 0.017) [13]. A recent study in a German population produced similar results, where the loss-of-function mutations in the *FANCM* gene were associated with both familial breast cancer and TNBC [14].

c.5791C>T mutation has also been identified in two colorectal cancer patients [17], and since FANCM is functionally connected with the mismatch repair genes *MSH2/MSH6*, it has previously been considered as a potential candidate gene for hereditary non-polyposis colorectal cancer (HNPCC) [17, 18]. However, a combined analysis of larger datasets did not reveal statistically significant association between *FANCM* mutations and the disease [19].

Here, we have investigated the association of the *FANCM* c.5791C>T mutation with breast cancer risk in familial and unselected breast cancer cases among 4806 invasive breast cancer patients and 2734 healthy population controls from four different geographical areas of Finland. We further evaluated the breast cancer risk by subgroups of patients as well as ovarian cancer risk among 526 ovarian cancer patients. Also, the recently identified c.4025_4026delCT (p.Ser1342*) and c.5293dupA (p.Thr1765Asnfs*3) variants in the *FANCM* gene were studied among 862 familial breast cancer patients from the Helsinki area.

Moreover, to further study the risk associated with truncating C-terminal *FANCM* mutations, we analyzed the current results of the c.5791C>T mutation and risk estimates of the c.5101C>T mutation, including the previously published results from Helsinki and Tampere area [12] and genotyping results from Oulu and Kuopio.

## Methods

### Subjects

#### Helsinki breast cancer series

The *FANCM* c.5791C>T mutation was genotyped in 2391 breast cancer cases (including unselected and familial patients) and 1258 healthy female population controls from the Helsinki area. Unselected breast cancer patient samples were collected at the Helsinki University Central Hospital in two phases. During 1997–1998 and 2000, 884 samples were collected at the Department of Oncology. These samples include 79% of all consecutive, newly diagnosed breast cancer cases during the collection periods [20, 21]. During 2001–2004, 986 samples, including 87% of all consecutive, newly diagnosed breast cancer cases were collected at the Department of Surgery [21]. Only invasive cases were included in this study.

The familial cases were collected at the Helsinki University Central Hospital Departments of Oncology and Clinical Genetics [22, 23]. Of these, 523 patients had a strong family history with at least three breast or ovarian cancers among first- or second-degree relatives (including the proband), and 555 patients had at least one first-degree relative affected with breast or ovarian cancer. All the familial cohort patients with at least three breast or ovarian cancers among first- or second-degree relatives were tested negative for *BRCA1/2* mutations and the patients with one affected relative were tested negative for the Finnish *BRCA1/2* founder mutations as previously described [24–26]. Eight hundred and sixty two familial cases were genotyped for the c.4025_4026delCT and c.5293dupA variants and 1078 familial cases were genotyped for the *FANCM* c.5791C>T mutation.

The samples are genomic DNA isolated from peripheral blood. The patient genealogies were confirmed from the population registries or hospital records and cancer diagnoses from the hospital records or the Finnish Cancer Registry. Hormone receptor status was collected from pathology reports as described earlier [27]. The 1258 genotyped population controls were healthy female blood donors from the Helsinki area.

The unselected ovarian cancer cohort was collected at the Helsinki University Central Hospital Department of Obstetrics and Gynecology in 1998 as previously described [28]. Blood samples were collected from invasive epithelial ovarian carcinoma treated patients during routine follow-up visits to the clinic. Additional samples were also collected between 1998 and 2006. Out of the 526 independent samples included in the analysis in this study, 408 were genomic DNA isolated from blood and 118 samples were tumor-DNA. Of the genomic samples, 171 were serous, 64 mucinous, and 43 endometrioid subtype. 129 samples were other ovarian cancer subtypes, for one sample the subtype is not known. Out of the tumor samples, 104 were serous and two were other subtypes, 12 samples were of unknown subtype.

#### Tampere breast cancer series

The unselected breast cancer patient sample set from the Tampere area was collected at the Tampere University Hospital as previously described [20, 22]. Additional 336 incident cases were collected in 1996–2004 at the Tampere University Hospital. Two hundred and forty nine cases had also familial background. Hormone receptor status information was obtained from patient pathology reports. Only invasive cases were included in this study. All samples are genomic DNA isolated from peripheral blood. The control cohort for the Tampere dataset consists of 808 healthy female blood donors from the Tampere area.

#### Kuopio breast cancer series

430 patients with invasive breast cancer from the Kuopio area were genotyped for the *FANCM* c.5791C>T and c.5101C>T mutations. These patients belong to prospective population-based case–control study, The Kuopio Breast Cancer Project (KBCP), which was conducted in between 1990 and 1996. Samples were collected from women entering the Kuopio University Hospital due to breast symptoms and who were eventually diagnosed as having breast cancer. All samples are genomic DNA isolated from peripheral blood. Information about hormonal receptor status was obtained from hospital registries [29, 30]. The control cohort consists of 158 healthy blood donors from the Kuopio area.

#### Oulu breast cancer series

The *FANCM* c.5791C>T and c.5101C>T mutations were screened among 1323 breast cancer cases (1147 unselected, 153 familial, and 56 young breast cancer patients) and 510 healthy female controls from the Oulu area. The unselected breast cancer samples were collected from patients operated at the Oulu University Hospital during 2000–2014, and they were unselected for age at disease onset and a family history of cancer. Only invasive cases were included in this study. The familial breast cancer cases were affected index individuals of Northern Finnish breast or breast and ovarian cancer families, and the young cohort consisted of breast cancer patients unselected for family history of cancer but with early disease onset (≤40 years), which suggests a plausible hereditary predisposition regardless of the family history [31–33]. Only *BRCA1/2* mutation negative familial and young cases were included in this study. Hormone receptor status was collected from pathology reports as described earlier [34]. The control cohort consisted of 510 healthy female blood donors from the Oulu area. All samples were genomic DNA isolated from peripheral blood.

This study was performed with informed consent from all the patients and permission from the ethics committees of University of Helsinki, Tampere University Hospital, Oulu University Hospital, University of Eastern Finland, and Kuopio University Hospital Board on Research Ethics.

### Identification of the mutations

The *FANCM* c.5791C>T mutation has been found to associate with familial breast cancer and TNBC [13, 14]. It has also been reported in the Finnish population as an enriched loss-of-function mutation [3] and identified in gene panel sequencing of Finnish familial breast cancer patients [35].

The c.4025_4026delCT variant was identified at Lund University in one patient from 100 Finnish high-risk breast cancer cases with panel sequencing, in which also two carriers of the previously published c.5101C>T mutation were identified. SureselectXT Custom 3-5.9 Mb library kit (Agilent Technology) was used to capture DNA fragments from the target genes. Sequencing was performed on the Illumina HiSeq 2500 with 2 × 94 to 2 × 101 bp paired-end reads.

The c.5293dupA variant was identified by exome sequencing of genomic DNA samples from 44 Finnish cancer patients with familial history of breast cancer. Exome sequencing was executed at the Genome Quebec Innovation Centre. To capture the exomic regions, Roche Nimblegen SeqCap EZ Exome v3 kit was used. The sequencing was performed on Illumina HiSeq 2000 sequencer with 100 bp paired-end reads.

The c.5101C>T mutation was identified by exome sequencing as previously described [12].

### Genotyping

Genotyping of the *FANCM* c.5791C>T mutation for the Helsinki and Tampere sample sets was performed with Sequenom MassARRAY system using iPLEX Gold assays (Sequenom) at FIMM (University of Helsinki). Variants c.4025_4026delCT and c.5293dupA were genotyped with TaqMan real-time PCR. 7500 Fast Real Time system was utilized by using TaqMan SNP Genotyping Custom assays and TaqMan Genotyping MasterMix (Applied Biosystems). Genotype calling was performed with 7500 RealTime PCR System and 7500 software (version 2.06, Applied Biosystems). Genotyping for the *FANCM* c.5101C>T mutation was performed as previously described [12]. Positive controls were used in all analyses and all mutations were confirmed with Sanger sequencing. It is to be noted that one patient from the Helsinki dataset (counted as one in the analysis) carries both c.5101C>T and c.5791C>T mutations. Unfortunately, we were not able to determine whether the mutations are in cis or in trans; however, if they were in cis, this genotype would be extremely rare.

*FANCM* c.5791C>T and c.5101C>T screening for Oulu and Kuopio sample sets was performed using High Resolution Melt analysis (CFX96, Bio-Rad) with Type-it HRM reagents (Qiagen). Positive control DNA was included in all analyses, and samples with positive-like or differing melting curves were validated by Sanger sequencing (ABI3130xl, Applied Biosystems).

### Statistical analyses

All statistical analyses were performed using R (version 3.02) statistical software (http://www.r-project.org) or IBM SPSS Statistics for Windows, version 22.0. For the risk analyses, two-sided P-values were calculated using Pearson´s *χ* ^2^-test or Fisher´s exact test if the expected number of cell count was five or less. *P* < 0.05 was considered statistically significant. Betas and standard errors of the different studies were combined in “rmeta”-package to examine heterogeneity between studies. In heterogeneity analysis, *P* < 0.10 was considered statistically significant.

In the combined analyses, all the datasets were pooled and the odds ratios and *P*-values were estimated with logistic regression model stratified by study. Separate analysis were conducted for the subgroups defined by histopathology and family history of the disease. In the combined analysis including datasets for both *FANCM* c.5791C>T and c.5101C>T mutations, patients with genotyping results for both mutations were included in the analysis.

## Results

### Genotyping of the *FANCM* c.5791C>T mutation in the case–control sample sets

The *FANCM* c.5791C>T mutation was identified in altogether 28 breast cancer patients and eight controls among 4806 breast cancer patients and 2734 population controls from four different geographical areas of Finland (Helsinki, Tampere, Kuopio, and Oulu). The population frequency was highest in Northern and Eastern Finland (Oulu 0.6% and Kuopio 0.6%) and lowest in Southern and Southwestern Finland (Helsinki 0.2% and Tampere 0.1%). Among the *BRCA1/2*-negative familial patients from Helsinki and Oulu datasets (*N* = 1231), eight mutation carriers were identified, and among the 526 ovarian cancer patients from Helsinki, two mutation carriers were identified (Table 1).

**Table 1:** Frequency of the *FANCM* c.5791C>T mutation in the studied sample sets by breast cancer subgroups and in the population controls

Study cohort	*N*	CC (%)	CT (%)	OR	95% CI	*P* value
Helsinki						
Controls	1258	1255 (99.8)	3 (0.2)	–	–	–
All BC	2391	2379 (99.5)	12 (0.5)	2.11	0.59–7.49	0.24
Unselected BC	1699	1690 (99.5)	9 (0.5)	2.23	0.60–8.25	0.22
Familial BC	1078	1073 (99.5)	5 (0.5)	1.95	0.46–8.18	0.48
≥3 affected	523	520 (99.4)	3 (0.6)	2.41	0.49–12.00	0.37
2 affected	555	553 (99.6)	2 (0.4)	1.51	0.25–9.08	0.64
ER+	1795	1786 (99.5)	9 (0.5)	2.11	0.57–7.80	0.25
ER−	412	409 (99.3)	3 (0.7)	3.07	0.62–15.26	0.16
TNBC	141	138 (97.9)	3 (2.1)	9.09	1.82–45.49	0.02
Unselected OC	526	524 (99.6)	2 (0.4)	1.60	0.27–9.58	0.64
Tampere						
Controls	808	807 (99.9)	1 (0.1)	–	–	–
All BC	662	657 (99.2)	5 (0.8)	6.14	0.72–52.70	0.10
ER+	494	490 (99.2)	4 (0.8)	6.59	0.73–59.11	0.07
ER−	122	121 (99.2)	1 (0.8)	6.67	0.41–107.34	0.25
TNBC	68	67 (98.5)	1 (1.5)	12.04	0.74–194.74	0.15
Oulu						
Controls	510	507 (99.4)	3 (0.6)	–	–	–
All BC	1323	1314 (99.3)	9 (0.7)	1.16	0.31–4.30	1
Unselected	1147	1141 (99.5)	6 (0.5)	0.89	0.22–3.57	0.87
Familial BC	153	150 (98.0)	3 (2)	3.38	0.68–16.92	0.14
YBR	56	56 (100)	0 (0)	–	–	–
ER+	434	432 (99.5)	2 (0.5)	0.78	0.13–4.70	1
ER−	109	108 (99.1)	1 (0.9)	1.56	0.16–15.19	0.54
TNBC	69	68 (98.6)	1 (1.4)	2.49	0.25–24.23	0.40
Kuopio						
Controls	158	157 (99.4)	1 (0.6)	–	–	–
All BC	430	428 (99.5)	2 (0.5)	0.73	0.07–8.15	1
ER+	318	317 (99.7)	1 (0.3)	0.50	0.03–7.97	1
ER−	95	95 (100)	0 (0)	–	–	–
TNBC	47	47 (100)	0 (0)	–	–	–

All BC: all genotyped breast cancer cases in designated study. ≥3 affected: families with three or more breast or ovarian cancer cases among first- or second-degree relatives. 2 affected: families with two first-degree relatives with breast or ovarian cancer

The patient subgroups are overlapping with 386 familial cases belonging also to the unselected cohort in the Helsinki data set and 33 familial cases belonging to the unselected cohort in Oulu data set. The patients may also belong to different subgroups by tumor phenotype

*BC* breast cancer, *OC* ovarian cancer, *ER* estrogen receptor, *TNBC* triple-negative breast cancer, *YBR* young breast cancer patients (age at diagnosis ≤40 years) among Oulu data set

We evaluated the breast cancer risk among all genotyped patients (all BC) in each dataset separately (Table 1). Furthermore, genotyped patients were divided into subgroups according to family history of cancer, ER status, and triple-negative subtype to study the risks by breast cancer phenotypes.

Breast cancer risk was increased among all breast cancer patients in the Helsinki (OR 2.11, 95% CI 0.59–7.49, *P* = 0.24), Tampere (OR 6.14, 95% CI 0.72–52.70, *P* = 0.10), and Oulu datasets (OR 1.16, 95% CI 0.31–4.30, *P* = 1), albeit with no statistically significant *P*-values. In the Kuopio dataset, only two mutations carriers were identified (OR 0.73, 95% CI 0.07–8.15, *P* = 1) (Table 1). No significant heterogeneity was seen between the studies (*P* = 0.9).

However, the *FANCM* c.5791C>T mutation associated significantly with TNBC in the Helsinki dataset (OR 9.09, 95% CI 1.82–45.49, *P* = 0.02). In the Tampere and Oulu datasets, two additional triple-negative cases were identified.

The analysis of ovarian cancer cases from the Helsinki dataset suggested possibly slightly increased risk among c.5791C>T carriers (OR 1.60, 95% CI 0.27–9.58, *P* = 0.64, Table 1); however, there were only two mutation carriers identified. One patient has serous ovarian cancer, whereas the other mutation carrier has been diagnosed with mucinous subtype of the disease.

We further performed a combined analysis of the four studies to evaluate the risk among all studied breast cancer patients. An elevated breast cancer risk for the c.5791C>T carriers was seen among all breast cancer patients (OR 1.94, 95% CI 0.87–4.32, *P* = 0.11), and particularly in the TNBC subgroup (OR 5.14, 95% CI 1.65–16.0), with a statistically significant *P* value (0.005) (Table 2).

**Table 2:** Combined analysis including all the sample sets (Helsinki, Tampere, Kuopio, and Oulu) in different subgroups, with number of the c.5791C>T mutation carriers and non-carriers

Study cohort	OR	*P*	95% CI	N Helsinki wt/mut	N Tampere wt/mut	N Oulu wt/mut	N Kuopio wt/mut
All BC	1.94	0.11	0.87–4.32	2379/12	657/5	1314/9	428/2
Familial BC	2.50	0.10	0.83–7.51	1073/5	–	150/3	–
Unselected BC	1.87	0.14	0.82–4.26	1690/9	657/5	1141/6	428/2
ER+	1.86	0.16	0.78–4.41	1786/9	490/4	432/2	317/1
ER−	2.34	0.14	0.75–7.35	409/3	121/1	108/1	95/0
TNBC	5.14	0.005	1.65–16.0	138/3	67/1	68/1	47/0

*BC* breast cancer, *ER* estrogen receptor, *TNBC* triple-negative breast cancer, *wt* wild type, *mut* mutation carrier

In addition, the breast cancer risk was increased also in the other subgroups, e.g., ER negative (OR 2.34, 95% CI 0.75–7.35, *P* = 0.14) and familial breast cancer (OR 2.50, 95% CI 0.83–7.51, *P* = 0.10) (Table 2), but the results did not reach statistical significance.

### Combined analyses of the *FANCM* c.5791C>T and c.5101C>T mutations

We combined the results from the current analyses of the *FANCM* c.5791C>T mutation with the previous risk study of the *FANCM* c.5101C>T mutation in the Helsinki and Tampere datasets [12], and genotyping results from Oulu and Kuopio cases and controls. Highly significant association was seen between breast cancer and carrying either of the mutations (OR 1.86, 95% CI 1.32–2.49, *P* = 0.0002). The risk was consistently increased in all subgroups of patients, with highest risk and the most significant association seen among the triple-negative patients (OR 3.08, 95% CI 1.77–5.35, *P* = 0.00007) (Table 3). No heterogeneity was seen between mutations (*P* = 0.7). All analyses were stratified by study.

**Table 3:** Combined analysis including all the sample sets (Helsinki, Tampere, Kuopio, and Oulu) in different subgroups, with the number of the *FANCM* c.5101C>T and c.5791C>T mutation carriers (combined) and non-carriers

Study cohort	OR	*P*	95% CI	N Helsinki wt/mut	N Tampere wt/mut	N Oulu wt/mut	N Kuopio wt/mut	N Helsinki controls wt/mut	N Tampere controls wt/mut	N Oulu controls wt/mut	N Kuopio controls wt/mut
All BC	1.86	0.0002	1.32–2.49	2285/81	656/35	1293/30	419/11	1234/21	782/21	498/12	157/1
Familial BC	1.99	0.004	1.24–3.19	1020/38	–	148/5	–	–	–	–	–
Unselected BC	1.77	0.0006	1.28–2.45	1652/58	656/35	1124/23	419/11	–	–	–	–
ER+	1.64	0.005	1.17–2.32	1721/55	480/22	426/8	292/8	–	–	–	–
ER-	2.02	0.004	1.25–3.25	405/16	114/8	108/1	99/2	–	–	–	–
TNBC	3.08	0.00007	1.77–5.35	130/10	60/5	68/1	58/2	–	–	–	–

*BC* breast cancer, *ER* estrogen receptor, *TNBC* triple-negative breast cancer, *wt* wild type, *mut* mutation carrier

### Genotyping of the *FANCM* c.4025_4026delCT and c.5293dupA variants

The *FANCM* c.5293dupA and c.4025_4026delCT variants were genotyped among 862 familial breast cancer patients from the Helsinki area. No additional mutation carriers were identified for c.5293dupA variant which may represent a unique mutation in the family where it was identified. The mutation was originally identified in exome sequencing of a uterine cancer patient with a family history of breast cancer.

One additional carrier was identified for c.4025_4026delCT, totaling two carriers for this rare variant and was not studied further. The patients´ ages at diagnosis were 42 and 54, respectively. One carrier had ER-negative breast cancer, whereas the other had ER-positive disease. Both had family history of breast cancer, but no additional samples were available for genotyping the relatives. The ExAC population frequency for the c.4025_4026 deletion in Finland is 0.015%.

## Discussion

In this current case–control study, we evaluated the breast and ovarian cancer risk for the *FANCM* c.5791C>T mutation as well as for c.4025_4026delCT and c.5293dupA variants among Finnish population. We further examined the risk associated with carrying either of the *FANCM* mutations c.5101C>T and c.5791C>T.

The study revealed the *FANCM* c.5791C>T mutation being more frequent among the studied breast cancer cases than in controls in the Finnish population. It was particularly enriched among the TNBC cases, showing a significant association in the combined analysis (OR 5.14, *P* = 0.005). This observation is consistent with previous studies on *FANCM* and breast cancer risk, as the c.5101C>T mutation has also been found to associate with TNBC [12, 14]. Consistent results were seen also in the other subgroups studied (Table 2).

We further combined the results of the *FANCM* c.5101C>T risk analysis [12] with the current results of c.5791C>T to study the risk associated with carrying either of these C-terminal *FANCM* mutations. The risk was significantly increased in all subgroups of patients, especially among triple-negative cases (OR 3.08, 95% CI 1.77–5.35, *P* = 0.00007).

Studying the c.5791C>T mutation in the ovarian cancer cases showed an elevated risk (OR 1.60), but the association was not statistically significant. Previous studies on *FANCM* mutations have shown similar results [12, 14]; however, a very recent study found significant association between *FANCM* mutations and high grade serous ovarian cancer [36].

Screening of the other two *FANCM* variants exposed only one additional c.4025_4026delCT carrier and no c.5293dupA carriers among 862 familial breast cancer patients, and these were not studied further. However, their identification may suggest a wider mutation spectrum of very rare mutations in the *FANCM* gene, potentially relating to subtype-specific breast cancer predisposition.

Of all breast cancers, about 10–20% are found to be hormonally triple-negative and these are usually aggressive with a poor prognosis, as this subtype does not respond to hormonal therapy. These tumors are often higher grade and larger size than the other tumor subtypes [37]. Germline mutations in *BRCA1* are common in TNBC cases, but deleterious changes in other homologous recombination DNA repair pathway genes have also been observed to occur relatively frequently in these patients, including *BRCA2*, *PALB2*, *BARD1*, and *RAD51C* [38]. The FA pathway has also been connected to TNBC, as comparison of mRNA expression in different breast tumor types revealed several FA pathway genes (*BRCA1*, *FANCD2*, *FANCF*, and *PALB2*) being significantly less expressed in TNBC tumors compared to luminal A (ER or PR positive) tumors [39]. This further supports the current finding that also FANCM depletion is linked especially with TNBC, but deeper understanding of this connection warrants further investigations. However, as the developmental mechanisms of TNBC are not well known, the identification of additional risk factors for this breast cancer subtype is valuable for understanding its etiology and for the identification of potential therapeutic targets.

FANCM has a crucial function in the DNA damage response for ICLs. It acts as a helicase/translocase and binds adjacent to the crosslink, inducing the recruitment of the FA core complex to the site. The core complex monoubiquitinates the FANCI–FANCD2 complex which triggers the accumulation of multiple nucleases and initiates the actual DNA repair processes [2]. Depletion of FANCM has been reported to have effects on both the FA pathway efficiency and tumorigenesis: FANCM deficient mouse embryonic fibroblasts displayed increased chromosomal breakage, residual FANCD2 monoubiquitination, and increased spontaneous sister chromatid exchanges, and the FANCM knockout mice had reduced overall and tumor-free survival [40].

The functional effect of *FANCM* c.5791C>T has previously been studied by Peterlongo et al. [13]. Instead of being a conventional nonsense mutation, it was proposed to create a binding site for a splicing factor hnRNP A1, which causes exon 22 skipping in mRNA and introduces a premature stop codon, leading to loss of 132 amino acids from the C-terminus of the protein. Two domains with important roles in binding of DNA, ERCC4 and helix–hairpin–helix (HhH)2, are located in the C-terminus of FANCM [41], and loss of them likely disturbs FANCM activity. Supporting this hypothesis, when the *FANCM* c.5791C>T mutation was introduced to mouse embryonic fibroblasts, the mutant cells displayed decreased DNA repair activity and increased chromosomal breakage [13]. This is consistent with the effect of the majority of the known breast cancer-associated gene mutations [42], further supporting the impact of *FANCM* c.5791C>T on genomic instability and increased cancer risk.

Altogether, the c.5791C>T mutation has to date been connected to familial breast cancer [13], and also to TNBC [14]. The current results on *FANCM* c.5791C>T, together with those of c.5101C>T, support a role for *FANCM* as a moderate-risk breast cancer susceptibility gene and further emphasize its connection with the triple-negative breast tumors.

## Conclusions

The current results provide further support for the previously suggested association between *FANCM* mutations and triple-negative breast cancer. Further studies with larger datasets are needed for precise risk estimations. Also, other rare variants may occur in the *FANCM* gene and warrant further investigations in other populations.

## Abbreviations

FA: Fanconi anemia
ICL: Interstrand crosslink
OR: Odds ratio
CI: Confidence interval
TNBC: Triple-negative breast cancer
ER: Estrogen receptor
PR: Progesterone receptor
HNPCC: Hereditary non-polyposis colorectal cancer
